# Enhanced Proliferation of Monolayer Cultures of Embryonic Stem (ES) Cell-Derived Cardiomyocytes Following Acute Loss of Retinoblastoma

**DOI:** 10.1371/journal.pone.0003896

**Published:** 2008-12-10

**Authors:** Satoshi Yamanaka, Ihor Zahanich, Robert P. Wersto, Kenneth R. Boheler

**Affiliations:** 1 Laboratory of Cardiovascular Science, National Institute on Aging, Baltimore, Maryland, United States of America; 2 Resource Research Branch, National Institute on Aging, Baltimore, Maryland, United States of America; University of Minnesota, United States of America

## Abstract

**Background:**

Cardiomyocyte (CM) cell cycle analysis has been impeded because of a reliance on primary neonatal cultures of poorly proliferating cells or chronic transgenic animal models with innate compensatory mechanisms.

**Methodology/Principal Findings:**

We describe an *in vitro* model consisting of monolayer cultures of highly proliferative embryonic stem (ES) cell-derived CM. Following induction with ascorbate and selection with puromycin, early CM cultures are >98% pure, and at least 85% of the cells actively proliferate. During the proliferative stage, cells express high levels of E2F3a, B-Myb and phosphorylated forms of retinoblastoma (Rb), but with continued cultivation, cells stop dividing and mature functionally. This developmental transition is characterized by a switch from slow skeletal to cardiac TnI, an increase in binucleation, cardiac calsequestrin and hypophosphorylated Rb, a decrease in E2F3, B-Myb and atrial natriuretic factor, and the establishment of a more negative resting membrane potential. Although previous publications suggested that Rb was not necessary for cell cycle control in heart, we find following acute knockdown of Rb that this factor actively regulates progression through the G1 checkpoint and that its loss promotes proliferation at the expense of CM maturation.

**Conclusions/Significance:**

We have established a unique model system for studying cardiac cell cycle progression, and show in contrast to previous reports that Rb actively regulates both cell cycle progression through the G1 checkpoint and maturation of heart cells. We conclude that this *in vitro* model will facilitate the analysis of cell cycle control mechanisms of CMs.

## Introduction

The regenerative capacity of adult mammalian heart is insufficient to restore cardiac function following serious injury. The theory of cardiac self-renewal has led to several lines of investigation to improve the clinical outcome of patients with damaged myocardium [1]–[3]. One research avenue involves use of autocrine or paracrine factors to limit cell death, modulate the migration or activities of inflammatory and/or cardiomyogenic cells, and improve blood flow. This approach may reduce inflammation, limit scar formation, improve vascularization and help preserve or restore function. In cases where damage has already occurred, cell transplantation has emerged as a reparative strategy, but the optimal source of cells remains to be defined. Derivatives of embryonic and adult stem or progenitor cells with cardiomyogenic potential have been proposed, but their propensity to generate multiple cell types and the inability to isolate primary cells without intervening cultivation has complicated their use [3]–[6]. Another option involves activation of cardiomyocyte (CM) or precursor cell proliferation [7], [8]. Although evidence for endogenous proliferation in adult heart exists, most cells are thought to be mitotically quiescent and activation is insufficient for repair [9]–[11]. Considerable effort has therefore been invested in the study of CM cell cycle regulation, but with limited success.

Because no proliferating cell line sufficiently close to normal cardiac muscle cells has ever been available, attempts to generate proliferating CM to study cell cycle progression have generally relied on primary cultures and expression of transforming oncogenes, the utility of which is limited by their transformed phenotype. Investigators have also employed late fetal and neonatal (and some adult) rat CM after infection with recombinant adenoviruses to alter principal cell cycle regulators [7]; however, adenovirus gene transfer vectors can induce cell cycle dysregulation and inappropriate expression of cyclin proteins, which complicate the interpretation of published studies [12]. Transgenic mouse models have also been extensively employed but are subject to developmental compensation or even embryonic lethality [7]. Use of inducible systems has helped eliminate these problems, but mouse models are time-consuming and expensive [13]. Embryonic stem (ES) cell derived cardiomyocytes (CM) have been proposed as an alternative model system to study CM cell cycle regulation [14], but to date, these experiments have only involved heterogenous cultures with unpurified cells.

From these diverse studies, it is generally accepted that over-expression of molecules (cyclins D1, D2, D3, cdk2 or E2F transcription factors) that promote progression through the G1 cell cycle restriction checkpoint can promote DNA synthesis [7], [15]–[19]; however, cell cycle progression in mature non-proliferating CM can lead to apoptosis [17], [20]–[23]. The role of retinoblastoma (Rb) protein in CM proliferation and apoptosis remains particularly enigmatic. In general, Rb is critical for G1 checkpoint control and is thought of as a negative regulator of cellular proliferation [24]. In G1 phase cells, Rb-histone deacetylase repressor complexes bind to E2F-dimerization partners 1 or 2 and other transcription factors to inhibit downstream transcription. Phosphorylation of Rb by CDK4 or 6 and CDK2 dissociates the Rb-repressor complex to release nuclear proteins, like E2F transcription factors, to permit transcription of S-phase genes [25]. MacLellan et al. were however unable to show an uncompensated role for Rb in fetal mouse heart. Cardiac-restricted Rb null animals did not display any defects in cardiac function or differentiation, and no differences in heart size, cell numbers, viability or lifespan could be demonstrated when compared to wild type animals. Similarly, mice in which the pocket protein p130 was knocked out showed no cardiac defects, but when p130 and Rb were both eliminated, significant changes in cell number and cardiac morphology were observed [26]. These data suggest that loss of one pocket protein could be compensated by protein redundancy within a cell. Because Rb is poorly expressed in fetal heart and upregulated during neonatal development [27], [28], some researchers have therefore suggested that Rb's primary role must be in terminal differentiation of CM [7]. More recently, however, Puceat found in Rb-deficient CM derived from ES cells, beating activity (EBs) is delayed in embryoid bodies and expression of GATA, MEF2 and Nkx-2.5 cardiogenic factors and myocardium-specific genes are downregulated [29], [30]. This led to the implication of LEK1 in cardiopoiesis and suggested that Rb played a more substantial role in early cardiomyogenesis than previously believed.

In the present study, we have addressed this paradox using the distal upstream region of the cardiac-restricted sodium-calcium exchanger 1 (*ncx1*) gene promoter [31], [32] in conjunction with mouse ES cells to isolate developmentally immature and actively proliferating CM that can be grown as a monolayer *in vitro*. We show that Rb, independent of both p107 and p130, acts both as a principal regulator of cell cycle progression through G1 and as a modulator of the transition from immature to mature CM.

## Results

### Characterization of ncx1-PAC resistant ES Cells

**Figure 1:**
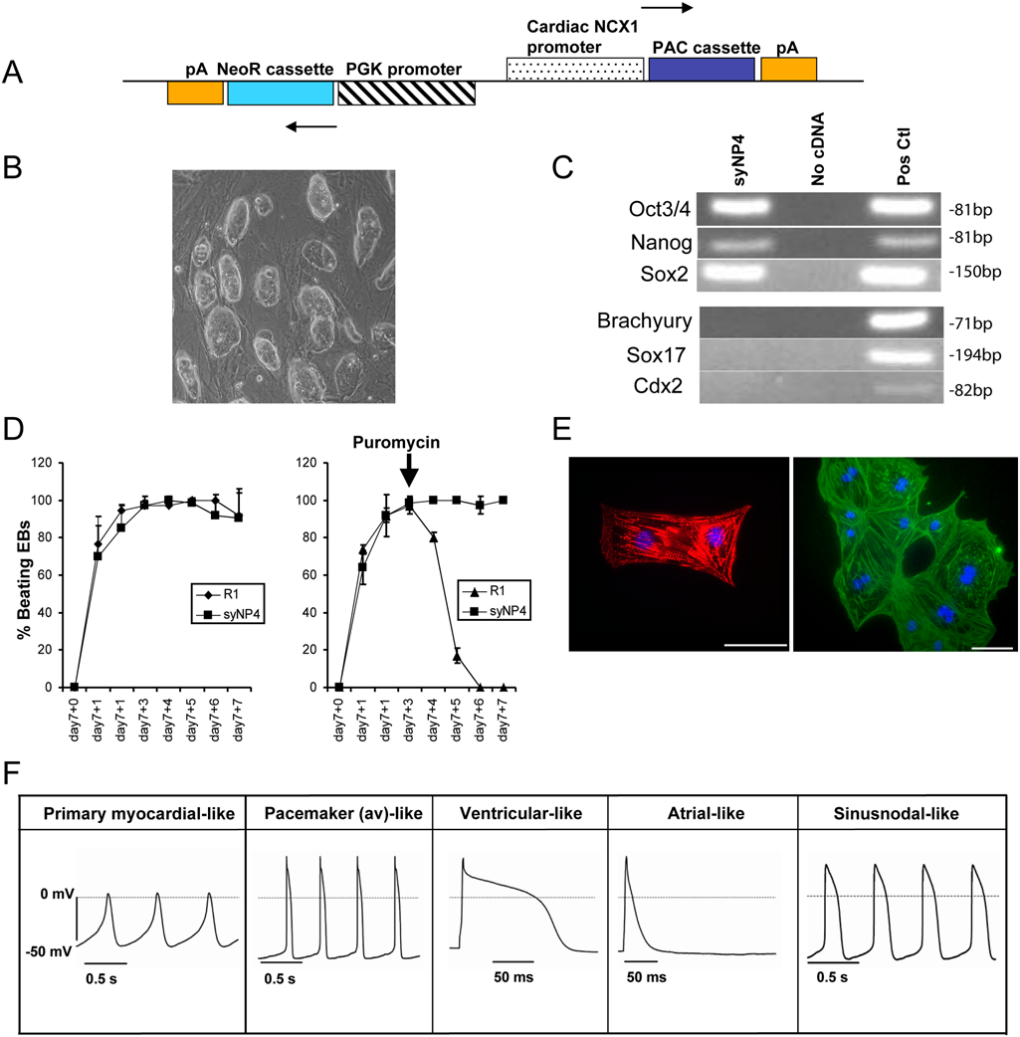
Generation and characterization of *ncx1*-PacR ES cells. A) Line drawing of *ncx1*-PAC/PGK-neoR plasmid construct introduced into parental R1 ES cells. B) Image of syNP4 ES cell colonies cultivated on feeder layers. C) Undifferentiated parental R1 and clonal ES cells lines express pluripotency marker RNAs for Oct3/4, Nanog and Sox2, but not for the mesodermal marker Brachyury, the endodermal marker Sox17 and the trophoectodermal marker Cdx2. Positive controls were from ES cells or EBs at days 4 and 6. D) Differentiation of ES cells led to the spontaneous induction of beating areas of CM (n = 24/experiment), and the differentiation capacity of syNP4 clones did not differ from R1 cells. Following puromycin (2.5 µg/mL) selection from D7+3, only syNP4 clones maintained contracting cells. Values are given as mean±SE from three independent experiments. E) Image of isolated puromycin-selected cells immunostained with antibodies against either α-actinin (left panel) or cTnT (right panel) and counterstained with Hoechst33324. Bar = 50 µm. F) Electrophysiological analysis of syNP4 cell derived CM, which differentiated into both immature and mature types of CM.

A linearized expression vector construct, containing a cardiac-restricted *ncx1* promoter driven puromycin resistance cassette and a phosphoglycerate kinase (*pgk*) promoter driven neomycin resistance (neoR) cassette, was electroporated into mouse R1 ES cells (Figure 1A). Clonal ES cell lines that randomly, but stably incorporated the linearized construct were expanded following selection. All of the G418-resistant clonal lines grew as compact colonies on feeder layers, and each colony was composed of cells with a high nuclear to cytoplasmic ratio (Figure 1B). Three ES clonal cell lines (syNP4, 13 and 34) were subjected to a comprehensive clonal analysis. All of these clonal lines stained positive for alkaline phosphatase activity, and expressed high levels of the pluripotency markers Oct4, Sox2 and Nanog. By PCR, Sox1, a marker of neuroectoderm, was just detectable, but Sox17, a pan-endoderm marker, Brachyury, a marker of early gastrulation and mesoderm differentiation, and Cdx2, a trophectoderm associated transcription factor, were undetectable in both undifferentiated R1 and G418 resistant clonal line (Figure 1C).

Parental R1 and G418 resistant clonal lines were used to generate CM through the formation of EBs via the hanging drop technique. With this method, all of the ES cell lines gave rise to beating areas within one day of EB plating (Figure 1D). Unlike syNP13 and 34, syNP4 routinely gave rise to beating clusters of cells in >90% of the plated EBs almost identical to that seen with the parental R1 line. Puromycin (2.5 µg/mL), an aminonucleoside antibiotic, was added at various concentrations and times to EBs to determine the most efficient selection protocol. When added prior to Day 7 (D7), no cells from either the parental R1 or clonal lines survived, indicating that the *ncx1* promoter was not activated prior to CM commitment and differentiation. When puromycin was added to cells after plating (D7+1 or 7+2), no contracting areas were ever observed 3 days after selection in R1 derived CM; however, when the clonal lines were examined, numerous foci with beating areas were readily observed (Figure 1D, right panel). To confirm that the cells were CM, puromycin-resistant cells were fixed and stained with antibodies to muscle-specific sarcomeric α-actinin and to cardiac specific troponin T (cTnT). In both instances, the antibodies stained the sarcomeric structures of all the cells examined (Figure 1E).

Because clone syNP4 had the best efficiency of CM differentiation relative to the other clonal lines, isolated single cells from this clone were analyzed electrophysiologically. Immature and spontaneously contracting CM were observed between D7+2 to 7+4; whereas, later stage cells (7+5 to 7+11) (n = 4 preparations) displayed multiple types of specialized action potentials (APs), which were either spontaneously activated or could be stimulated with injected current (Figure 1F). Specific cell types that could be observed consisted of primary myocardial-like cells (7 of 8 early stage cells) and more mature CM with pacemaker-like activities or adult-like atrial and ventricle-like cells. Early and late CM spontaneous contractile activity differed in the values of maximum diastolic potential (MDP), AP amplitude and upstroke velocity. Some of the spontaneously beating cells from later stages (3 out of 24 cells) displayed an intermediate shape of APs (represented by a presence of the transient component, MDP around −70 mV and AP amplitude over 90 mV). Late pacemaker-like CM with sinus node-like APs responded to 20 µM carbachol, a muscarinic antagonist, whereas early CM did not. After application of carbachol, spontaneous activity of nodal-like CM was strongly suppressed or completely abolished. By comparison, atrial- and ventricular-like types of AP differed in their shape of plateau and AP duration at 50% of repolarisation. Based on the membrane potential before activation, the AP duration, measured as the time period from depolarisation to 50% repolarisation (APD_50%_), the presence of a long AP plateau and shape of the AP (dome- or triangle-like, typical for ventricular- and atrial-like CM, respectively), and the presence or absence of carbachol-sensitivity, these cells have been sub-divided into 5 groups (Table 1). Importantly, the type and frequency of CM was almost identical to that observed with wild-type ESCM generated from R1 or D3 ES cells [33].

**Table 1:** Electrophysiological properties of ESCM isolated at D7+2–4 and D7+5–11.

Cell type	V_m_ (mV)	APD_50%_ (ms)	Amplitude (mV)	Number of cells (n = 31)
**Primary Myocardial-like**	−47.16±4.47	152.46±19.1	71.05±8.07	10 (32%)
**Atrial-like**	−66.41±3.32	31.89±4.92	90.47±6.03	7 (22%)
**Ventricular-like**	−72.65±1.39	80.9±14.99	90.92±4.01	5 (17%)
**Sinusnodal-like**	−67.8±1.71	70.69±18.22	90.35±2.23	5 (17%)
**Pacemaker (AV)-like**	−68.53±1.54	53.66±10.06	98.98±3.97	4 (12%)

Specialization of the cells was based on the action potential parameters: resting membrane potential (V_m_), AP duration at 50% of repolarisation (APD_50%_) and amplitude of the AP. Shown are data of primary myocardial-like, atrial-, ventricle- and sinus node-like cells. At D7+2–4, 7 of 8 cells displayed features typical of immature primary myocardium and one cell was AV-like; whereas, between D7+5–11, all but three cells displayed a more mature phenotype typical of atrium, ventricle and nodal regions (sinus and atrio-ventricular).

### Highly Purified Early Stage Cardiomyocytes Derived From ES cells

**Figure 2:**
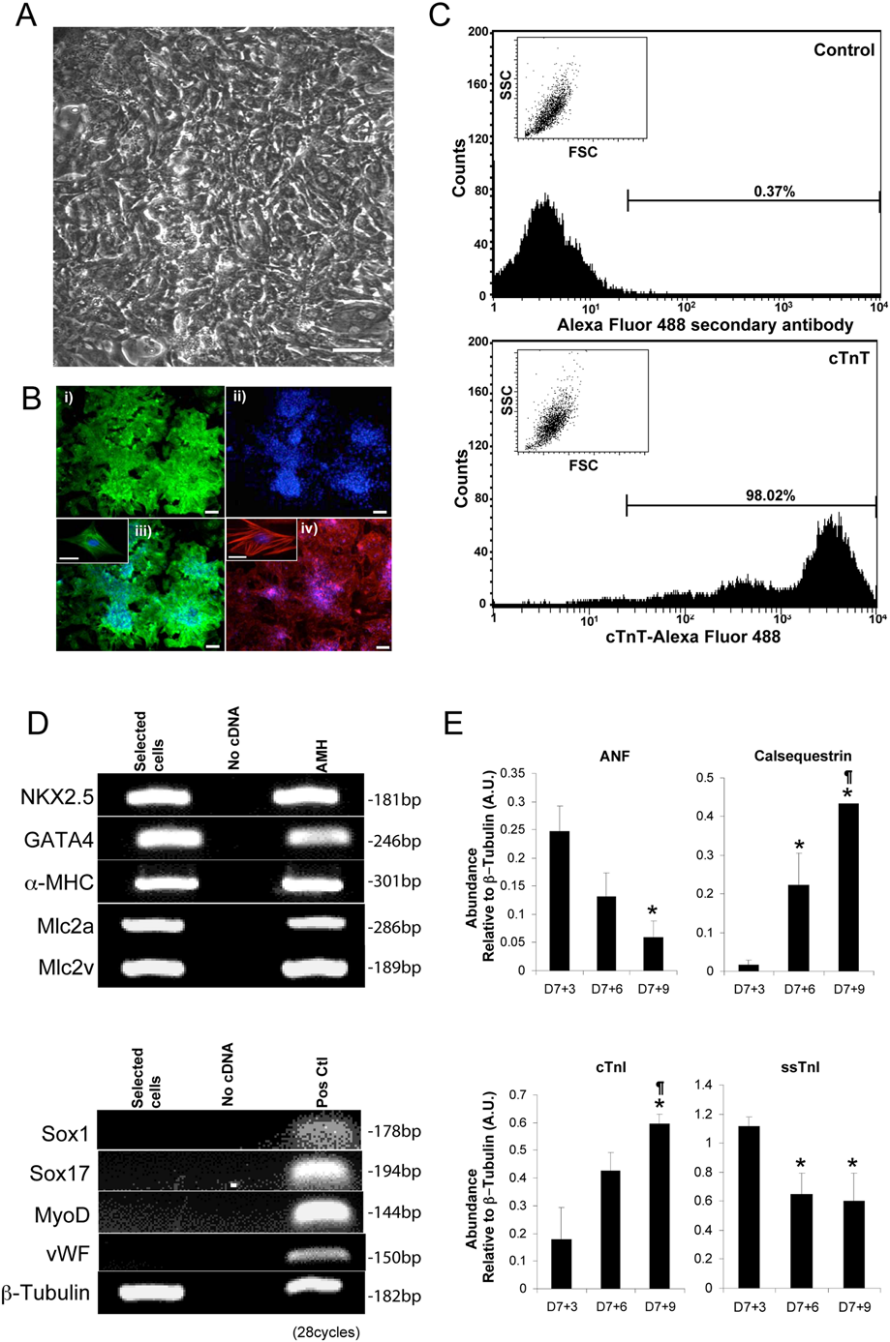
Monolayer cultures of highly purified ESCM. A) Bright field image of puromycin-resistant ESCM at D7+9. In this example, D7+1 EBs were collagenase treated and replated in the presence of puromycin for 3 days. Bar = 50 µm. B) Selected cells (D7+4) were stained with Hoechst 33342 (ii, iii and vi) and probed with antibodies to either α-actinin (i and iii) or cTnT (iv). Magnified views (inset) show individually stained CM. Bar = 100 µm (Insert = 30 µm). C) Puromycin resistant cells (D7+3) were probed for cTnT and analyzed by flow cytometry. The results showed that >98% of the ungated cells in the monolayer culture were positive for cTnT. Insets show forward and side scatter plots of analyzed cells (linear scale). D) Transcripts to cardiac-associated Nkx2.5, Gata4, α-MHC, Mlc2a and Mlc2v were highly abundant, but those to Sox1, Sox17, MyoD and von Willebrand factor (vWF) were not detected in any of the pooled RNA samples from monolayer cultures. E) QRT-PCR analysis of gene transcripts differentially expressed during CM maturation, showing a significant increase in calsequestrin and decrease of atrial natriuretic factor (ANF) as a function of time. Slow skeletal (ss) isoforms of TnI were also temporally replaced by cTnT.

*W*e optimized a 6-step protocol to isolate a highly purified population of proliferating CM from syNP4. Using the hanging drop technique and induction with ascorbate, both individual and multiple foci of spontaneously contracting areas were observed within one day of EB plating. To maximize cell purity, EBs (D7+1) were digested with collagenase to obtain single cells or small clusters of cells. This heterogeneous cell population was replated, and within 24–48 hours of puromycin selection, a monolayer culture of contracting cells was observed (Figure 2A). Within 3 to 6 days, cell density increased to cover >70–90% of the plate surface. At this time, most if not all of the cells were immunopositive for α-actinin and cTnT (Figure 2B).

To quantitatively determine the number of CM in the culture following puromycin selection, we analyzed the cells by flow cytometry (Figure 2C). We determined that >98% of the fixed cells were immunopositive for cTnT. Although the remaining <2% might represent “contaminating non-CM”, some of these may have been due to incomplete cell permeabilization and an inability of the anti-cTnT antibody to bind its antigen. By RT-PCR, we determined that cardiac-associated transcripts to Nkx2.5, Gata4, α-myosin heavy chain (MHC), myosin light chain (Mlc) 2a and Mlc2v were abundant. Smooth muscle alpha actin RNA, a marker of both smooth muscle cells and immature or de-differentiated CM was detectable [34], [35], but transcripts encoding Sox1 (neuroectoderm), Sox17 (pan-endoderm), skeletal muscle-specific MyoD and endothelial cell-specific von Willebrand factor were not detectable in any pooled sample (Figure 2D). With increased cultivation times, RNAs encoding cardiac calsequestrin increased [33], [36], but a decrease in transcripts encoding atrial natriuretic factor (ANF) was observed [37] (Figure 2E). The slow skeletal (ss) isoform of TnI was also temporally replaced by the cardiac isoform [38], [39].

**Figure 3:**
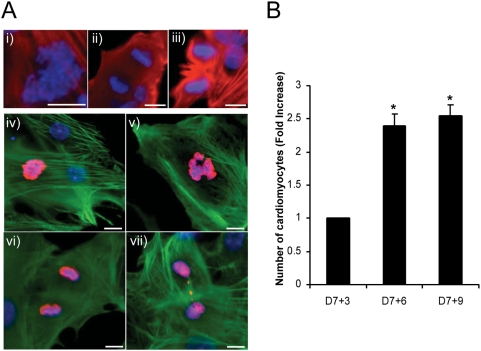
Puromycin selected ESCM proliferate *in vitro*. A) Images of CM in various stages of mitosis. Puromycin-resistant cells were stained with Hoechst 33342 (blue), and probed with antibodies specific to cTnT (red; i–iii, green; iv–vi) and phospho-H3 Histone (red; iv-vi). In examples i-iii, condensed chromatin, metaphase and telophase were observed at D7+3–4. In iv–vii, mitotic activity was confirmed by staining with an antibody to phospho-H3 Histone, which is only phosphorylated at Ser10 during mitosis. Mitotic nuclei are pink (red+blue). Bar = 10 µm. B) Graph showing that CM numbers increase by 2.4±0.2-fold between D7+3 and 7+6 (*, P<0.005).

Perhaps more importantly, mitosis was readily observed in fixed, cTnT labelled cells during the first three days of selection. Mitotic cells with condensed chromatin, in early and late metaphase, and in telophase were all observed (Figure 3A). Mitotic activity was further confirmed by staining with an antibody that only reacts with Histone H3 when it is phosphorylated at Ser10 during mitosis. Moreover, monolayer cultures of CM established with this protocol demonstrated a significant 2.4±0.2-fold increase in cell number between D7+3 and 7+6 (Figure 3B, p<0.05). No further increase in the number of cells occurred between D7+6 and 7+9, and it was almost impossible to find mitotic cells in late cultures (D7+9 or 15).

**Figure 4:**
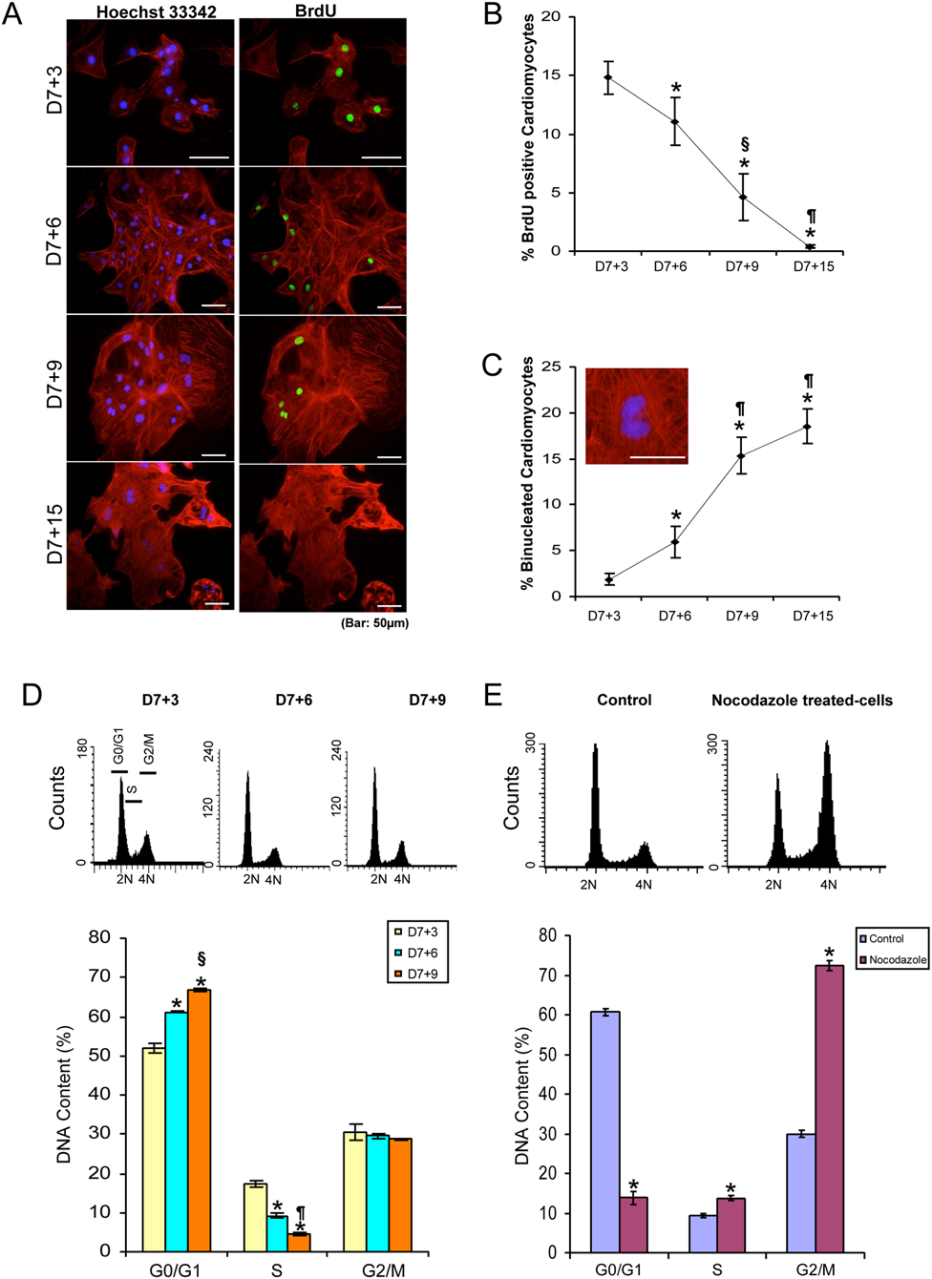
Early stage CM actively progress through the cell cycle. A) BrdU positive (green, S phase), cTnT positive (red) cells were routinely observed at early time points after selection, but only rarely at late time points. Bar = 50 µm. B) Graph depicting the percentage of BrdU labeled CM as a function of time (*, P<0.005 vs D7+3, §, P<0.05 vs D7+6, ¶, P<0.005 vs D7+6, ∫, P<0.05 vs D7+9). C) Graph showing the percentage of binuclear CM stained with both cTnT and Hoechst 33324 (Bar = 50 µm) between D7+3 and 7+15 (*, P<0.005 vs D7+3, §, P<0.05 vs D7+6, ¶, P<0.005 vs D7+6, ∫, P<0.05 vs D7+9). D) DNA content was measured on PI-labelled fixed cells by flow cytometry, and the relative positions of G0/G1, S and G2/M phase cells are shown. The graph indicates the number of cells in each phase of the cell cycle as a function of time (*, P<0.05 vs D7+3, §, P<0.05 vs D7+6, ¶, P<0.05 vs D7+3). E) DNA content analysis of 120 hour nocodazole-treated cells and percentage of cells in each phase of the cell cycle. *, P<0.005 versus control.

The DNA content of monolayer cultures as a function of cultivation time was analyzed. First, selected ESCM were pulse labelled with BrdU for 1 hour followed by fixation and co-staining with antibodies to BrdU and to cTnT (Figure 4A). BrdU is a thymidine analog that is actively incorporated into DNA of replicating cells, and is a reliable index of cells in S phase. In these experiments, the percentage of BrdU positive, cTnT positive CM at D7+3 was 14.8±1.4%, but this percentage decreased to 0.4±0.1% by D7+15 (p<0.005, Figure 4B). During this time frame, a significant increase in the number of binuclear CM was observed. The percentage of binucleated CM was initially very low, but it increased from 1.9±0.7% at D7+3 to 15.4±2.0% at D7+15 (p<0.005, Figure. 4C). The DNA content of propidium iodide (PI) labelled cells showed a significant decrease in the number of S phase cells (D7+3:17.3±0.7%; D7+6: 9.2±0.7%; D7+9: 4.4±0.5%) concomitant with an increase of the number of cells in the G0/G1 phase of the cell cycle (D7+3: 52±1.2%; D7+6: 61.2±0.03%; D7+9: 67±0.4%) (Figure 4D). No significant change in the percentage of cells in the G2/M phase could be demonstrated at any time point examined. This latter result is attributable, at least in part, to binucleation. Thus, some ESCM proliferate in early cultures, but with continued cultivation time, cells stop dividing and actively undergo a maturation process characterized by binucleation concomitant with changes in gene expression (e.g., calsequestrin, ssTnI, cTnI and ANF).

To determine the percentage of CM actually progressing through the cell cycle and not in G0, D7+1 selected cells were treated with nocodazole, a drug that selectively blocks cells in mitosis by inducing microtubule dynamic instability (Figure 4E, top). In pilot experiments, we determined that 100 ng/mL of nacodazole was effective at blocking ESCM in mitosis. In untreated cells 62±2.1% were in G0/G1, but 48 hours after the addition of nocodazole, the percentage decreased to 45.4±1.6%. This reduction decreased further to 25.1±1.2% at 72 hours post-treatment. At 120 hours post-treatment, less than 13.8±1.5% of the cells were in either G0 or G1 (Figure 4E, bottom). Concomitant with this time dependent decrease in G0/G1 cells, a significant increase in the number of cells in G2/M was observed, such that at 120 hours post-nocodazole treatment, 86.2±1.5% of CM were either in the S or G2/M phase of the cell cycle (n = 4). Since the starting number of binucleated cells was very low and binucleation would not be expected following nocodazole blockade in G2, we conclude that the maximum number of ESCM in G0 (or G1) is less than 15% of the total and that a majority of early CM (>85% from nocadozole) are actively progressing through the cell cycle.

### Role of pRb in early ES cell-derived cardiomyocytes

**Figure 5:**
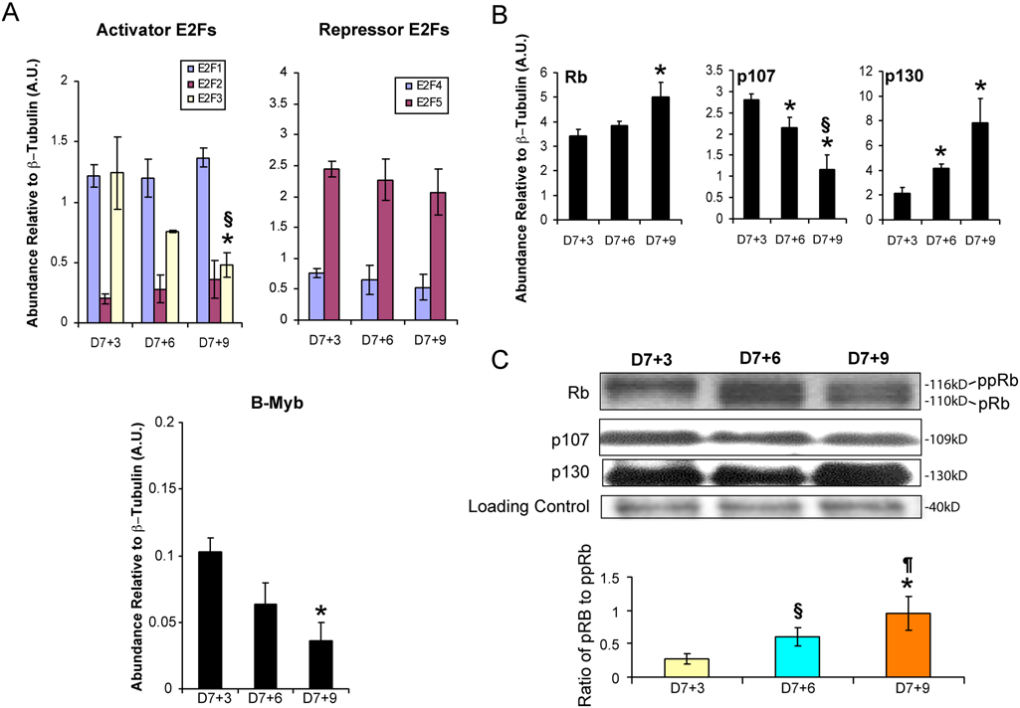
Regulators of G1 cell cycle progression are dynamically regulated in early ESCM. A) QRT-PCR analysis of activator and repressor E2F and B-Myb associated transcripts. (*, P<0.05 vs D7+3, §, P<0.05 vs D7+6). B) Relative abundance (qRT-PCR) of pocket proteins (Rb, p107, p130) as a function of time (*, P<0.05 vs D7+3, §, P<0.05 vs D7+6). C) Western showing the abundance of pocket proteins in ESCM and the phosphorylation state of Rb (*, P<0.0005 vs D7+3, §, P<0.005 vs D7+3, ¶, P<0.05 s D7+6) . Relative abundance of hypo- (pRb) to hyperphosphorylated (ppRb) is shown graphically.

Members of the transcription factor E2F gene family are determinants of G1/S-phase transitions during the mammalian cell cycle. Activator E2Fs are maximally expressed late in G1 and are generally found in association with E2F regulated promoters during the G1/S transition (e.g., B-Myb); whereas, inhibitors are generally expressed in quiescent cells (G0) in association with E2F-binding elements on E2F-target promoters during G0-phase. Activator E2F activity is directly regulated by the action of retinoblastoma (Rb) protein and indirectly through the action of G1 cyclins and associated kinases (CDK4/6, CDK2). The abundance of five E2F gene transcripts (Activators: E2F1, 2 and 3a; Repressors: E2F4 and 5) was therefore examined by qRT-PCR. Of activator E2Fs, only E2F3a demonstrated a significant change in abundance with time (Figure 5A), suggesting that this transcription factor may play a crucial role in the proliferation and maturation of early ESCM. Transcripts encoding repressor E2F4 and 5 were observed, but no difference in expression could be demonstrated between D7+3 and 7+9. Since inhibitor E2Fs 3b, 6, 7 and 8 were not measured, it is possible that some cells might have stopped proliferating because of entry into G0. B-Myb gene transcription is however normally activated very late in G1 and is necessary for the regulation of S phase gene expression [40]. Since it's expression decreases as a function of cultivation time, the data indicate that ESCM must either be actively withdrawing from the cell cycle or undergoing a checkpoint delay in G1.

To assess the latter possibility, expression of pocket proteins (pRb, p107, and p130) were examined. Although RNA expression of Rb and p130 increased and p107 decreased as a function of time (Figure 5B), at the protein level only Rb protein showed significant changes (Figure 5C). The hypo-phosphorylated or active (inhibitory) form of Rb markedly increased as a function of *in vitro* cultivation time. On Westerns, this transition could be seen by loss of the upper hyper-phosphorylated band, whereas the lower band represents hypophosphorylated forms of Rb. In these experiments, the ratio of hypo-Rb to hyper-Rb increased by almost 4-fold and ranged from 0.27±0.08 at D7+3 to 0.95±0.26 at D7+9, consistent with activation of G1 checkpoint controls.

**Figure 6:**
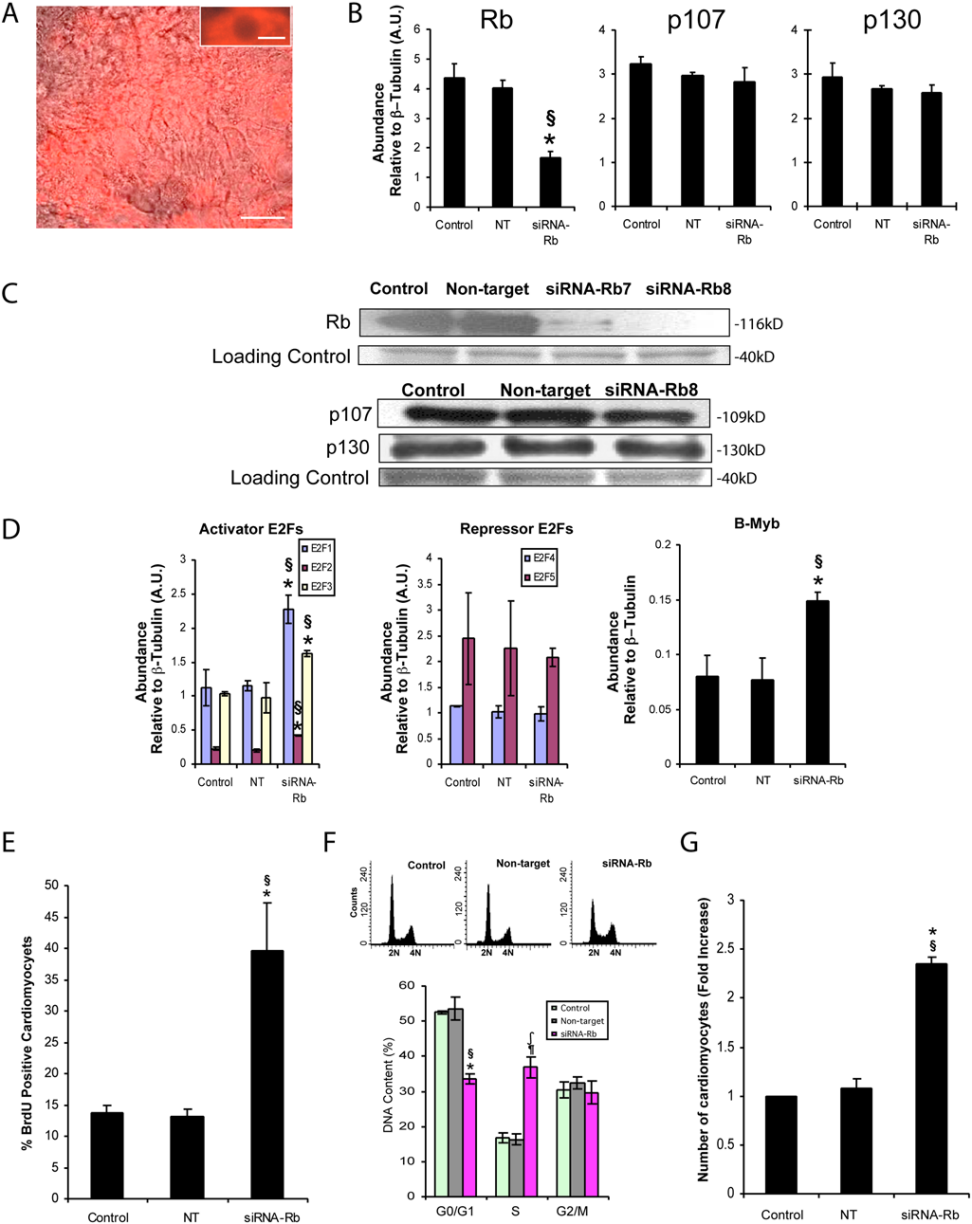
Knockdown of Rb alters the expression of factors implicated in the G1 to S phase transition. A) Image of purified ESCM transfected with siGlo RNA showing that >90% of the puromycin-resistant cells were transfected. Bar = 50 µm (Insert = 10 µm). B) QRT-PCR analysis of Rb, p107 and p130 mRNA in purified ESCM transfected with siRNAs against Rb (*, P<0.05 vs control, §, P<0.05 vs non-target). C) Western blot images showing Rb, p107 and p130, following transfection with siRNA-Rb8. Loading control (actin) image was altered from a bitmap image, due to the loss of the tiff file. D) QRT-PCR analysis of E2F1-E2F5 and B-Myb, following KD of Rb (*, P<0.05 vs control, §, P<0.05 vs nontargeting, ¶, P<0.005 vs control). E) Percentage of BrdU labeled CM (cTnT positive) after KD of Rb in puromycin selected cells (b, *, P<0.0005 vs control, §, P<0.0005 vs nontargeting). F) Cell cycle distribution (DNA content of PI-stained nuclei) of cells after KD of Rb relative to controls (*, P<0.0005 vs control, §, P<0.05 vs nontargeting, ¶, P<0.005 vs Control, ∫, P<0.005 vs nontargeting). g) Graph showing CM numbers following siRNA-Rb mediated KD of Rb in puromycin-resistant cells (*, P<0.0005 versus control, §, P<0.0005 versus nontargeting).

SiRNAs were also employed to specifically knockdown (KD) Rb. As controls, experiments were performed with a nontargeting siRNA and with siGlo as a fluorescent indicator of the number of transfected cells. Transfection with siGlo showed that >90% of purified early CM (D7+4) were successfully transfected (Figure 6A), similar to what we previously reported in neonatal rat CM [41]. In these experiments, no significant difference in Rb transcripts could be demonstrated between nontargeting siRNA transfected cells and nontransfected cells (Figure 6B). Four different siRNA-Rbs were examined and based on pilot experiments, transfection with siRNA-Rb7 and siRNA-Rb8 significantly reduced Rb transcripts in early CM within 48 hours compared to transfection with a nontargeting siRNA or no transfection control. The most efficient reduction of Rb transcripts was in cells transfected with siRNA-Rb8, and only these data are shown unless indicated. No significant difference in p107 and p130 expression could be demonstrated at any timepoint examined following KD of Rb; however, Rb transcripts were decreased by >60%. Comparable results for each pocket protein were observed on Western blots (Figure 6C), except that the Rb protein was decreased to a greater extent (80–90%) than the RNA. Thus expression of Rb could be specifically reduced without any changes in expressions of p107 and p130.

**Figure 7:**
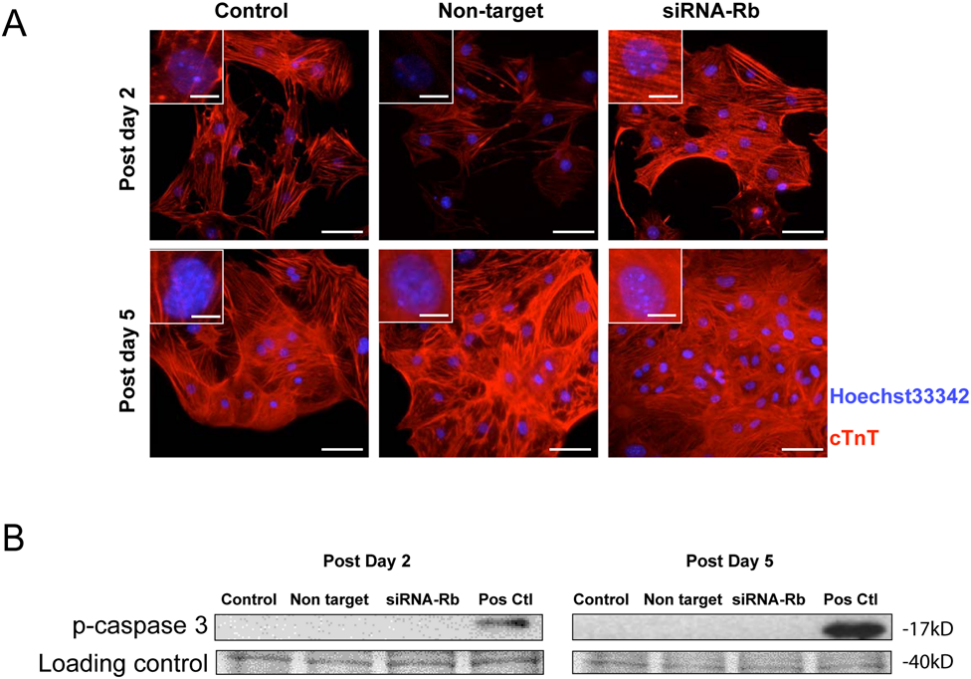
Knockdown of Rb in proliferating CM does not induce apoptosis. A) CM were stained with cTnT and Hoechst 33342 at two time points, D2 and 5, after transfection with siRNA-Rb. The appearance of condensed or fragmented nuclei and activated caspase 3, as an index of apoptosis, was then assessed. In these experiments, no altered nuclear fragmentation or condensation was observed among any of the groups, following KD of Rb. Magnified views (inset) show nuclei stained with Hoechst 33324 without characteristics of apoptosis. Bar = 50 µm (Insert = 10 µm). B) Western blot showing phosphocaspase-3, which was used as an indirect measure of apoptosis.

Knockdown of Rb dramatically altered expression of numerous factors implicated in cell cycle progression without an induction of apoptosis. E2F1, E2F2, E2F3a and B-Myb transcripts significantly increased. In contrast, no significant change in E2F4 and E2F5 could be demonstrated between siRNA-Rb8 transfected and control transfected cells (Figure 6D). Moreover, the percentage of BrdU positive CM increased from 13.7±1.3% in controls to 39.6±7.7% (n = 4, Figure 6E). Flow cytometric analysis of PI labelled cells furthermore demonstrated a significant >2-fold increase in the number of S phase cells concomitant with a significant decrease in G0/G1, but not G2/M cells (Figure 6F). The total CM numbers increased by 2.4±0.1%-fold following KD of Rb (Figure 6G). Apoptosis, which can occur in adult CM induced to proliferate, was also assessed. In these experiments, where over 100 cells were evaluated from independent fields, no significant difference in the number of nuclei with characteristics consistent with apoptosis (DNA condensation and fragmentation) could be demonstrated in control versus Rb KD cells (Figure. 7A). Consistently, no phospho (active) caspase-3 was detected in any group at any time point examined (Figure. 7B). Thus, KD of Rb, without compensatory changes of expressions in p107 and p130, led to the up-regulation of E2F-1, -2, -3a and B-Myb, which in turn induced DNA synthesis and proliferation without induction of apoptosis.

## Discussion

The G1 cell cycle checkpoint controls passage of cells from the first gap (G) phase into the DNA synthesis (S) phase. Cell cycle checkpoint controls are regulated, in part, by protein kinases (cyclin-dependent kinases or CDKs) and activating partners (cyclins) to ensure that each phase of the cell cycle (G1, S, G2, M) is complete prior to progression into the next phase. A transcription factor complex that includes members of retinoblastoma pocket protein (Rb, p107 and p130) and E2F (E2F1-5) gene families and two cyclin-cell cycle kinase complexes (CDK4/6-cyclin D and CDK2-cyclin E) are pivotal to the control of the G1/S cell cycle checkpoint, but in heart the relatively late up-regulation of Rb in development and its lack of effect on cardiac cell numbers in knockout mice have led investigators to conclude that the primary role of Rb involves terminal differentiation of CM. Here, we show that Rb also plays a critical role in ESCM cell cycle progression, thus expanding the list of modulators directly implicated in CM progression through G1.

Klug et al. were the first to suggest that ESCM might provide a useful *in vitro* system to evaluate CM cell cycle regulation [14]. Since then, the field of stem cell differentiation has made important strides, but little attention has been focused on the cell cycle. Several groups reported isolation of ESCM with cardiac gene promoters, including *nkx2.5*, cardiac α-*mhc*, *mlc2v*, or cardiac α-actin. Developmentally, each promoter exhibits a unique pattern of expression that is more or less suitable for analysis of cell cycle progression. The transcription factor NKX2.5 is one of the earliest cardiac markers. It has been employed to identify a progenitor cell population that can give rise to multiple cell types, but it's expression is not restricted to CM [42], [43]. In rodent heart development, α-MHC is only weakly expressed in the primary myocardium before it becomes restricted to atrial regions throughout the remainder of embryonic and early fetal development [44]. It is therefore most useful for the preferential selection of ES-derived atrial cells [45]. The *mlc2v* promoter has been used to identify “ventricular” myocytes from differentiating ES cells *in vitro* [46], but its “specific” expression is only apt for adult rodent heart. During development, it's expression is restricted to anterior (atrial and atrio-ventricular) portions of the heart tube, and at later stages, in the caval myocardium [44], [47]. The activity of the cardiac α-actin gene promoter is also robust in heart, but it is expressed in skeletal muscle cells particularly during embryogenesis [48], [49].

In contrast, we showed that the distal upstream region of cardiac restricted *ncx1* promoter is cardiac restricted during embryonic and fetal development [31], [50], and others have reported that this *ncx1* promoter confers the highest degree of cardiac specificity compared to other cardiac specific genes [51]. It is uniformly expressed in the heart starting in the cardiac progenitors cell containing cardiogenic plate region at ∼7.75dpc [31], and it is active in proliferating myocardium, including the poorly differentiated but highly proliferative compact zone. Perinatally, it's expression decreases as CM withdraw from the cell cycle [31], [50]. We were also successful at using the distal sodium-calcium exchanger promoter with a neomycin resistance cassette to isolate more mature, non-dividing ESCM [32], and in the present study, we report that it can be employed to isolate actively proliferating CM.

Maximal activation of the distal upstream portion of the sodium-calcium exchanger 1 gene requires GATA4/6 and NKX2.5 [31]. Since NKX2.5 expression identifies a cardiac progenitor cell population [42], [43], regulation of this upstream portion of the sodium-calcium exchanger 1 gene may be critical to the transition of cardiac progenitors from the primitive state to the pool of amplifying myocytes. Endogenous cardiac progenitors may however have cell cycle control mechanisms and gene markers that differ from the proliferating ESCM described in this paper. This is an important point because the generation of amplifying myocytes derived from the growth and differentiation of cardiac progenitor cells *in vivo* may fundamentally differ from the process whereby ES cells produce progeny *in vitro*. ES cells have a higher innate replicative capacity than most adult stem/progenitor cells, and ES cell cycle withdrawal is generally associated with differentiation [3]–[5]. In contrast, endogenous adult cardiac progenitor cells are thought to either proliferate at a relatively slow rate *in vivo* [9], [10] or alternatively these cells like those from the hematopoietic system may withdraw from the cell cycle until stimulated to divide and/or differentiate [3].

During the *in vitro* transition from a proliferative to non-proliferative state described here, we observed a number of characteristic molecular and functional changes. Specifically in puromycin-resistant CM, there is a switch from an immature cell type characterized by ssTnI and ANF expression to a more mature phenotype characterized by cTnI and calsequestrin gene expression. This is accompanied by a change from APs characterized by an immature (−40 mV) to intermediate resting membrane potential (−70 mV), an increased amplitude of depolarization, and an intermediate AP duration (45 msec), to APs more typical of atrial-like and ventricular-like cells. Importantly, the molecular phenotype and AP parameters of early ESCM are most similar to CM derived from early embryonic heart [32], [52], leading us to conclude that puromycin-selected cells derived from clone syNP4 at initial stages are proliferatively viable and relatively immature. Furthermore, CM show dynamic changes that affect regulatory proteins implicated in cell cycle progression. The expression of the activator protein E2F3a, but not E2F1 or E2F2, decreases significantly, consistent with reports showing an essential role of E2F3 in cardiac development [29], [53]. Expression of repressor E2Fs (E2F4 and E2F5) do not increase as proliferation decreases; however, there is a significant increase in the number of cells in G0/G1 [8]. Since at least 85% of the cells actively progress through the cell cycle, the vast majority of these early CM can not be in G0, and since there are initially high levels of B-Myb, we suggest that the primary delay in cell cycle progression is at a G1 checkpoint.

S phase cell numbers decrease significantly as a function of time, but no change in G2/M cell numbers was observed at any time point examined. This suggests that some cells might be blocked at a G2 checkpoint or had become binucleated and withdrawn from the cell cycle. Initially, we find that <2% of the proliferating cells are binuclear, but by D7+15, >15% of ESCM have this phenotype. This is consistent with the report by Klug et al. who showed that ESCM loose DNA synthetic capacity concomitant with the increase of the number of binuclear CM [14]. Soonpaa et al. also reported that *in vivo* cell cycle withdrawal was associated with the appearance of binucleated CM, most likely attributable to karyokinesis without cytokinesis [54]; and in the adult mouse heart, where contractile cells have withdrawn from the cell cycle, about 90% of CM are binuclear [54].

Zandstra et al. purified a large number of CM from ES cell lines carrying a neoR cassette driven by the α-MHC gene promoter from EBs cultivated in a stirred suspension culture system. Similar to our report with G418 resistant ESCM [32], it took several days to achieve a high purity of CM [55]. Although no specific analysis of the cell cycle was performed in Zandstra's study, at least some of these cells continued to proliferate even at late time points [55]. In the present study, selected ESCM actively proliferated for only about 3 to 6 days, before a significant reduction in proliferation occurred. These results are in agreement with those of Klug et al. who showed that non-purified ESCM were mitotically active at D11 (equivalent to D7+4 in this study), but at D21, mitotic activity was present in <1% of cells [14]. Apparent differences in proliferation among these studies might be due to culture conditions. In our system and that of Klug et al., CM were cultivated as adherent cells, and as cell densities increase with proliferation, some essential growth factor may become limiting. Alternatively adherent cells may be subject to contact inhibition that might not be seen in suspension. If the latter were involved, then one would expect a concomitant increase in the expression of the CDK inhibitors p21 and p27, which negatively regulate progression through restriction point in G1 phase. In fact, expression of p21 and p27 gradually increases concomitant with the decrease of proliferative capacity during cultivation as adherent cells (unpublished data, SY).

In this study, we observed that Rb goes from a hyperphosphorylated to a hypophosphorylated form with time of cultivation, and most of the cells appeared to be delayed in G1. To determine if Rb were actively implicated in control of a specific G1 checkpoint, we employed RNAi's to acutely KD this protein. Based on a variety of controls, the >60% reduction in Rb mRNA expression was both rapid and specific (it did not cause a reduction in either p107 and p130), and it promoted CM proliferation. In fact, the number of CM more than doubled, BrdU incorporation tripled and all three activator E2F transcription factors were up-regulated. B-Myb expression was also elevated, without any change in repressor E2Fs. These data indicate that cells actively cycled through the G1 checkpoint when Rb protein levels were reduced. Since a reduction in markers of maturation (e.g. increased ANF and decreased calsequestrin) was also observed, Rb must have a dual role in proliferation and in maturation. These latter effects may however be secondary to changes in proliferation and total cell numbers. While these data differ from those reported in mice [26], there is precedent to account for this disparity. Sage et al. reported that acute loss of Rb in mouse embryonic fibroblasts induced cell cycle re-entry and cell proliferation; whereas germline loss of Rb did not. These differences were explained by a functional compensation with other pocket proteins (p107) present in animal models that did not occur in the cells [56]. We therefore propose a similar non-compensatory mechanism to account for differences observed between our results and those of MacLellan et al. [26].

Consistent with this view, Rb is known to inhibit both cell division and apoptosis, but the choice often depends on the cell type as well as the amounts of Rb present in a cell [24]. Deletion or complete inactivation of Rb in some cancer cells can induce apoptosis without proliferation [57]. In contrast, MacPherson et al. reported that conditional central nervous system (CNS) loss of Rb through a Cre recombinase-loxP mediated event induced cell proliferation without apoptosis. In embryos, however, pronounced apoptosis was observed in CNS cells that fully lacked Rb [58]. In our experiments, we only achieved >60% and >80% reduction of Rb transcripts and proteins respectively, which resulted in CM cell proliferation without apoptosis. It remains unclear whether complete loss of Rb would lead to a different result, but it does show that an acute reduction or inactivation of Rb, independent of p107 or p130, promotes CM proliferation.

Finally, cell transplantation has emerged as a strategy to treat damaged myocardium, but the optimal cell source remains to be defined. Adult cardiomyogenic stem and progenitor cells have been proposed for cardiac regenerative medicine, but it remains unclear which multipotent stem or progenitor cell is best suited for transplantation and cardiac repair [4], [59]. Induced pluripotent stem (iPS) or embryonic stem (ES) cell progeny may represent viable alternatives, but only if issues associated with immunological rejection and possible tumor formation can be overcome [3], [4], [60]. Fully committed embryonic-like or early fetal-like CM derived from either embryonic or adult stem or progenitor cells may however prove optimal for cardiac transplantation. This is because fetal rat CM functionally integrate and couple with endogenous heart cells [61]; whereas, multipotent cells give rise to numerous types of progeny, including non-CM, some of which may not be apt for cardiac regeneration. In addition, cardiac cell transplantation is usually targeted to damaged myocardium with limited blood flow (ischemia) or poor oxygenation (hypoxia); consequently, mitochondria rich CM with high oxidative metabolism would not survive in such an environment. In contrast, immature (fetal) CM are tolerant of ischemia and have an innate ischemic preconditioning-like response mechanism [62]–[64]. Not only are early, immature CM hypoxia resistant, many of these cells retain the ability to proliferate [7], [8], which may make them ideal candidates for transplantation. Thus, this cell preparation has potential clinical implications. The ability to inhibit the pluripotency of ES cells and direct their linage commitment may become a powerful therapeutic strategy for human disease, and the use of proliferatively competent and fully committed ESCM may emerge as a viable source to treat damaged human myocardium.

In conclusion, we have described an *in vitro* model system that permits isolation of CM that transiently proliferate in monolayer cultures *in vitro*. We demonstrate that Rb does in fact play an active role in control of CM cell cycle progression, and in contrast to mouse models lacking Rb in heart, acute loss of Rb appears to be a central regulator of the G1/S transition in developmentally early stage CM, consistent with most reports on Rb in other non-cardiac systems. Based on these findings, we suggest that this model is directly applicable to the examination of molecular mechanisms controlling cell cycle regulation of early stage CM, and these studies may ultimately foster therapeutic interventions with proliferatively competent, fully committed CM derived from stem cells.

## Materials and Methods

### Generation of Transgenic ES Cell Clones

A plasmid carrying both the cardiac-restricted portion of the sodium-calcium exchanger 1 gene (*ncx1*) promoter driven puromycin resistant cassette (PacR) and a phosphoglycerate kinase promoter driven neomycin resistant transgene (*pGK*-NeoR) was constructed. The cardiac restricted *ncx1* promoter fragment consisted of a 2730-bp upstream region and 45bp of a cardiac-restricted untranslated exon [31]. The puromycin resistance cassette was cloned downstream of the untranslated exon. A *pGK*-NeoR-pA (1.7kb EcoRV and Sma1 fragment) from PGKneoKXRO was inserted into the multiple cloning site upstream of the most distal portion of the *ncx1* promoter in the reverse orientation. The transgene construct containing both the *ncx1*-PAC and *pGK*-NeoR sequences was isolated by digestion with BamH1 and Nsi1, and 50 µg of the fragment was transfected into the mouse ES R1 cell line via electroporation. Transfected clones were selected with G418 (300 µg/ml, Gibco/BRL), and the presence of both the *ncx1*-PAC and pGK-NeoR sequences in the resulting cell lines was confirmed by PCR. Three clones, syNP4, 13 and 34, of 112 G418-resistant colonies were selected for subsequent analyses. The clones were tested for the expression of pluripotency markers by PCR and endogenous alkaline phosphatase activity (Vector Laboratories).

### Culture, Differentiation of ES Cells and Generation of High Purified Cardiomyocytes

R1 and syNP (NP: *ncx1*,PacR) clonal lines were cultivated and differentiated, as described previously [65], [66], but to generate larger numbers of CM, ascorbate was added at D2 [67]. Briefly, undifferentiated ES cells were cultivated on mitomycin C-inactivated mouse feeder layers or cultured under feeder free conditions on 0.1% gelatin-coated tissue culture dishes in the presence of 1,000U/mL or 2,000U/mL, respectively of leukemia inhibitory factor (LIF) (Chemicon) containing Medium. Differentiation of ES cells into CM was initiated by a hanging drop technique to form EBs. Ascorbic acid (10^−4^M, Sigma) was added to the medium at D2 to augment the differentiation into CM. Seven days after formation, EBs were transferred to a 24-well 0.1% gelatin-coated dish. Total differentiation time is indicated as D7+*N,* where D7 (or earlier) indicates the time in suspension and N is the time after plating. Beating areas within plated EBs (*n* = 24) were counted daily and used as in indication of CM differentiation.

For the generation of highly purified early stage ESCM, EBs at D7+1 were digested with collagenase [65] for 8 minutes to generate small clusters of cells or single cells. Cells were pooled and resuspended in differentiation medium containing 2.5 µg/mL puromycin prior to plating onto multiple 0.1% gelatin-coated 6 or 10 cm dishes. Non-puromycin resistant cells are killed within 24–48 hours of selection. Cell numbers were determined with Vi-CELLTM XR according to the manufacture's protocol (BECKMAN COULTER), and equivalent numbers of cells were plated. The same instrument was employed to determine total CM cell numbers following selection from parallel plates seeded with the same starting, unselected cell population.

### Immunostaining and Image acquisition

syNP4 cell-derived CM were examined by immunostaining using primary antibodies to mouse α-actinin (Sigma, 1∶800, A7811) and mouse cardiac troponin T (cTnT, NeoMarkers, MS295R7), and rabbit histone H3 (phospho S10) (H3P, 1∶200, Abcam, ab5176). Briefly, cells were fixed in 2% paraformaldehyde in PBS, washed with PBS and permeabilized with 0.2% Triton X-100 (Sigma). Non-specific binding was blocked with a solution of 1% bovine serum albumin (BSA) in PBS, and cells were incubated with the primary antibody. After washing, cells were incubated with Alexa Fluor 488 or 568 conjugated goat anti-mouse IgG or Alexa Fluor 568 conjugated goat anti-rabbit IgG (Invitrogen). Hoechst 33342 (Molecular Probes, 5 µg/mL) was used to label nuclei. Images were obtained by fluorescence microscopy using a Zeiss Axiovert 35 microscope (West Germany) with Zeiss lenses (Plan–Neofluar, 63X/1.25 oil, 40X/1.30, 10X/0.25 and 5X/015) coupled to a SPOT Camera (Diagnostic Instruments, Inc). Image acquisition was obtained with SPOT Advanced, version 4.0.9 software. Following acquisition, tiff or jpeg files were placed in composite images with the assistance of Photoshop software. Image data were not manipulated in any manner in any of the figures, excluding the adjustment of contrast or brightness.

### Western Blotting

Western blotting was performed with 25 µg of protein resolved on a 5% or 4–15% gradient Tris-HCl gel and transferred to PVDF membranes. Rat neonatal fibroblasts treated with 10mM Camptothecin (Sigma-Aldrich) were used for a positive control for phospho caspase-3. Membranes were probed with primary antibodies to mouse Rb (1∶250, BD Bioscience, #554136), mouse p107 (1∶200, Santa Cruz, sc-250), rabbit p130 (1∶250, Santa Cruz, sc-317), and rabbit cleaved Caspase-3 (1∶500, Cell Signaling, #9661). Blots were then probed with HRP conjugated goat anti-rabbit IgG (H+L) or goat anti-mouse IgG (H+L) (Zymed) and horseradish peroxidase was detected using Pierce Super Signal ECL substrate kit and chemiluminescence captured on Kodak BioMax Light film. Band densitometry was performed using ImageQuant 5.1 image analysis software and either unmanipulated jpeg or tiff files were used in making the figure composites.

### Cardiomyocyte DNA Synthesis

DNA content was measured on propidium iodide (PI, Sigma)-stained nuclei from ice cold 70% ethanol fixed cells using a FACsCalibur as previously described [68]. Cell cycle compartments were deconvoluted from single-parameter DNA histograms of 10,000 cells. To block the CM in G2, purified syNP4 cell-derived CM were treated with 100ng/mL nocodazole (Sigma) for 24 to 120 hours. Cells were trypsinized (both the floating and the adherent cells were collected) and fixed with ice cold 70% ethanol. DNA cell cycle analysis was performed as described.

To determine the purity of selected CM-derived from clone syNP4, cells were harvested and trypsinized, and single-cell suspensions were fixed with an ice cold 1∶1 mixture of methanol/acetone. Primary anti-cTnT antibodies were incubated as described above, followed by incubation with a secondary Alexa Fluor 488 conjugated goat anti-mouse IgG. The number of labelled cells were determined with a FACsCalibur from 10,000 cells.

CM progression in S phase was quantified by 5-Bromodeoxyuridine (BrdU) (Sigma) incorporation into DNA as described previously [68]. Briefly, puromycin resistant cells were labeled with BrdU at a concentration of 10 µM for 1 hour. After BrdU incubation, cells were fixed and stained with an antibody to cTnT as described above. Cells were then incubated with 4N HCl/1% Triton X-100 to denature the DNA and extract histones. Cells were incubated with anti-BrdU ALEXA488 conjugated antibody (Molecular Probes/Invitrogen, A21303) followed by nuclear staining with Hoechst 33342. Each experimental condition was assayed in triplicate, and a minimum of 300 CM nuclei was counted. The results are reported as the mean percentage of BrdU positive CM nuclei.

### RT-PCR, Real Time PCR

RNA quantities were determined after reverse transcription using either standard or real-time PCR techniques as described previously [69]. Briefly, total cell RNA was extracted using Trizol (Invitrogen) followed by DNAse treatment. cDNA synthesis was performed with 500 ng of total RNA using a High Capacity cDNA Archive Kit (Applied Biosystems). Reverse transcriptase (RT) and real-time quantitative (Q) PCR reactions were performed with an ABI PRISM 7900HT Sequence Detector System (PE Applied Biosystems) using a SyberGreen Protocol in a 384 well plate format with the core reagent kit and the SYBR Green PCR Master Mix (Applied Biosystems). Primer sequences for RT and Q-PCR are shown in Table 2.

**Table 2:** Primers for PCR (* for q-PCR)

Transcript	Primer sequence (Forward/Reverse)	Tm	Product size
Oct3/4	CAATGCCGTGAAGTTGGAGA	60	81
	GCTTCAGCAGCTTGGCAAAC		
Nanog	TTTCAGAAATCCCTTCCCTCG	60	81
	CGTTCCCAGAATTCGATGCT		
Sox2	AAGGGTTCTTGCTGGGTTTT	60	150
	AGACCACGAAAACGGTCTTG		
Brachyury T	AGGCTCCCCTGCACATTACA	60	71
	AGCAGCCCCTTCATACATCG		
Cdx2	GGGTGGGGGTAGCAATACTT	60	82
	TGCCTCTGGCTCCTGTAGTT		
NKX2.5	CGACGGAAGCCACGCGTGCT	60	181
	CCGCTGTCGCTTGCACTTG		
GATA4	TCTCACTATGGGCACAGCAG	60	246
	CGAGCAGGAATTTGAAGAGG		
α-MHC	CTGCTGGAGAGGTTATTCCTCG	64	301
	GGAAGAGTGAGCGGCGCATCAAGG		
Mlc2a	CAGACCTGAAGGAGACCT	52	286
	GTCAGCGTAAAACAGTTGC		
Mlc2v	TGTGGGTCACCTGAGGCTGTGGTTCAG	60	189
	GAAGGCTGACTATGTCCGGGAGATGC		
Sox1	AGATGGCCCAGGAAAACCC	60	178
	CCTCGGACATGACCTTCCAC		
Sox17	TCCCTACCAGGGACACGACT	60	194
	GAGCTAGCGTCGGACACCAC		
MyoD	ATGCTGGACAGGCAGTCGAGGC	65	144
	GCTCTGATGGCATGATGGATTACAGCG		
vWF	CCACTTGCCACAACAACATC	60	150
	TGGACTCACAGGAGCAAGTG		
β-Tubulin	GAAGAGGAGGCCTAACGGCAGAGAGCCCT	60	182
	GAGTGCCTGCCATGTGCCAGGCACCATTT		
ANF*	TGATAGATGAAGGCAGGAAGCCGC	64	203
	AGGATTGGAGCCCAGAGTGGACTAGG		
Calsequestrin*	TGAACTTCCCCACGTACGATG	60	307
	AAACTCAATCGTGCGGTCACC		
cTnI*	AGGGCCCACCTCAAGCA	58	103
	GGCCTTCCATGCCACTC		
ssTnI*	GCACTTTGAGCCCTCTTCAC	60	169
	GTGTTCCTGCTCCCAACACT		
Rb*	CCTTGAACCTGCTTGTCCTC	60	191
	GGGCAAGGGAGGTAGATTTC		
P107*	GATGCTCATCTGACCGGAGT	60	83
	ATAAGTCACGTAGGCGCACA		
P130*	GGAAATGCCCTTCAGTGTTC	60	101
	GGACGCTTTAGAGTCCTTGG		
E2F1*	ATGGAAGAGGACCAACTGTC	60	230
	CCTGAATCCCTAGGCTTCTG		
E2F2*	TAGGGAGATGTGGAGGATTCGG	60	206
	AACTCAGGGTGGACAAACAAACAC		
E2F3a*	GCCTCTACACCACGCCACAAG	60	99
	TCGCCCAGTTCCAGCCTTC		
E2F4*	CTTCTACCTCCTTTGAGCCCATC	60	96
	TCACAGACACCTTCACTCTCGTCC		
E2F5*	ACCTGATGACCTCACACAGCCTTC	60	202
	GGGGTAGGAGAAAGCCGTAAAAG		

### RNA Interference of Rb Expression

Post-transcriptional gene silencing was performed using chemically synthesized duplex RNA oligonucleotides against mouse Rb (Catalog number LQ-047474-00-0005, Dharmacon, Lafayette, CO), as described previously [41]. Briefly, purified syNP4 cell-derived CM were placed in medium to which 100nM short interfering (si) RNAs was added along with the GeneSilencer reagent (GenLantis Corp., San Diego) for 24 hours. Four siRNA targeting Rb transcripts were tested, but only two were effective in pilot experiments. Experiments were performed both with a non-targeting siRNA and with siGlo (RFP) as a fluorescent indicator.

### Electrophysiological Analysis

APs in ESCM were studied during continuous superfusion with a solution containing 140 mM NaCl, 5.4 mM KCl, 5 mM HEPES, 2 mM MgCl_2_, 1.8 mM CaCl_2_, 10 mM glucose, (pH adjusted to 7.4 with NaOH). APs were measured at physiological temperature (35±0.5°C) by perforated patch-clamp technique (Axopatch 200, Axon Instruments, Foster City, CA, USA) with 50 µM β-escin [70] added to the pipette solution. pCLAMP7 software was used for data acquisition and analysis (Axon Instruments, Foster City, CA, USA). Electrode solution contained 120 mM K-gluconate, 5 mM NaCl, 5 mM Mg-ATP, 5 mM HEPES, 20 mM KCl, 3 mM Na_2_ATP (pH adjusted to 7.2 with NaOH).

### Statistical Analysis

Results are presented as mean±S.D. A Student's t test with repeated independent measurement was employed to determine statistical significance. P values less than 0.05 were considered as stastiscally significant. Correlation coefficients were determined using functions in Microsoft Excel.

## References

[1] Koh GY, Soonpaa MH, Klug MG, Field LJ (1995). Strategies for myocardial repair.. J Interv Cardiol.

[2] Rubart M, Field LJ (2006). Cell-based approaches for cardiac repair.. Ann N Y Acad Sci.

[3] Perino MG, Yamanaka S, Li J, Wobus AM, Boheler KR (2008). Cardiomyogenic stem and progenitor cell plasticity and the dissection of cardiopoiesis.. J Mol Cell Cardiol.

[4] Wobus AM, Boheler KR (2005). Embryonic stem cells: prospects for developmental biology and cell therapy.. Physiol Rev.

[5] Beltrami AP, Barlucchi L, Torella D, Baker M, Limana F (2003). Adult cardiac stem cells are multipotent and support myocardial regeneration.. Cell.

[6] Moretti A, Caron L, Nakano A, Lam JT, Bernshausen A (2006). Multipotent embryonic isl1+ progenitor cells lead to cardiac, smooth muscle, and endothelial cell diversification.. Cell.

[7] Bicknell KA, Coxon CH, Brooks G (2007). Can the cardiomyocyte cell cycle be reprogrammed?. J Mol Cell Cardiol.

[8] Ahuja P, Sdek P, MacLellan WR (2007). Cardiac myocyte cell cycle control in development, disease, and regeneration.. Physiol Rev.

[9] Anversa P, Leri A, Kajstura J, Nadal-Ginard B (2002). Myocyte growth and cardiac repair.. J Mol Cell Cardiol.

[10] Beltrami AP, Urbanek K, Kajstura J, Yan SM, Finato N (2001). Evidence that human cardiac myocytes divide after myocardial infarction.. N Engl J Med.

[11] Rubart M, Field LJ (2006). Cardiac regeneration: repopulating the heart.. Annu Rev Physiol.

[12] Wersto RP, Rosenthal ER, Seth PK, Eissa NT, Donahue RE (1998). Recombinant, replication-defective adenovirus gene transfer vectors induce cell cycle dysregulation and inappropriate expression of cyclin proteins.. J Virol.

[13] Yutzey KE, Robbins J (2007). Principles of genetic murine models for cardiac disease.. Circulation.

[14] Klug MG, Soonpaa MH, Field LJ (1995). DNA synthesis and multinucleation in embryonic stem cell-derived cardiomyocytes.. Am J Physiol.

[15] Soonpaa MH, Koh GY, Pajak L, Jing S, Wang H (1997). Cyclin D1 overexpression promotes cardiomyocyte DNA synthesis and multinucleation in transgenic mice.. J Clin Invest.

[16] Tamamori-Adachi M, Ito H, Sumrejkanchanakij P, Adachi S, Hiroe M (2003). Critical role of cyclin D1 nuclear import in cardiomyocyte proliferation.. Circ Res.

[17] Ebelt H, Hufnagel N, Neuhaus P, Neuhaus H, Gajawada P (2005). Divergent siblings: E2F2 and E2F4 but not E2F1 and E2F3 induce DNA synthesis in cardiomyocytes without activation of apoptosis.. Circ Res.

[18] Busk PK, Hinrichsen R, Bartkova J, Hansen AH, Christoffersen TE (2005). Cyclin D2 induces proliferation of cardiac myocytes and represses hypertrophy.. Exp Cell Res.

[19] Hassink RJ, Pasumarthi KB, Nakajima H, Rubart M, Soonpaa MH (2008). Cardiomyocyte cell cycle activation improves cardiac function after myocardial infarction.. Cardiovasc Res.

[20] Liu Y, Kitsis RN (1996). Induction of DNA synthesis and apoptosis in cardiac myocytes by E1A oncoprotein.. J Cell Biol.

[21] Kirshenbaum LA, Abdellatif M, Chakraborty S, Schneider MD (1996). Human E2F-1 reactivates cell cycle progression in ventricular myocytes and represses cardiac gene transcription.. Dev Biol.

[22] Agah R, Kirshenbaum LA, Abdellatif M, Truong LD, Chakraborty S (1997). Adenoviral delivery of E2F-1 directs cell cycle reentry and p53-independent apoptosis in postmitotic adult myocardium *in vivo*.. J Clin Invest.

[23] von Harsdorf R, Hauck L, Mehrhof F, Wegenka U, Cardoso MC (1999). E2F-1 overexpression in cardiomyocytes induces downregulation of p21CIP1 and p27KIP1 and release of active cyclin-dependent kinases in the presence of insulin-like growth factor I.. Circ Res.

[24] Sun A, Bagella L, Tutton S, Romano G, Giordano A (2007). From G0 to S phase: a view of the roles played by the retinoblastoma (Rb) family members in the Rb-E2F pathway.. J Cell Biochem.

[25] McClellan KA, Slack RS (2007). Specific *in vivo* roles for E2Fs in differentiation and development.. Cell Cycle.

[26] MacLellan WR, Garcia A, Oh H, Frenkel P, Jordan MC (2005). Overlapping roles of pocket proteins in the myocardium are unmasked by germ line deletion of p130 plus heart-specific deletion of Rb.. Mol Cell Biol.

[27] Flink IL, Oana S, Maitra N, Bahl JJ, Morkin E (1998). Changes in E2F complexes containing retinoblastoma protein family members and increased cyclin-dependent kinase inhibitor activities during terminal differentiation of cardiomyocytes.. J Mol Cell Cardiol.

[28] Jiang Z, Zacksenhaus E, Gallie BL, Phillips RA (1997). The retinoblastoma gene family is differentially expressed during embryogenesis.. Oncogene.

[29] Papadimou E, Menard C, Grey C, Puceat M (2005). Interplay between the retinoblastoma protein and LEK1 specifies stem cells toward the cardiac lineage.. Embo J.

[30] Puceat M (2005). Rb and LEK1: A “Pas de Deux” in Cardiogenesis.. Cell Cycle.

[31] Koban MU, Brugh SA, Riordon DR, Dellow KA, Yang HT (2001). A distant upstream region of the rat multipartite Na(+)-Ca(2+) exchanger NCX1 gene promoter is sufficient to confer cardiac-specific expression.. Mech Dev.

[32] Fijnvandraat AC, van Ginneken AC, Schumacher CA, Boheler KR, Lekanne Deprez RH (2003). Cardiomyocytes purified from differentiated embryonic stem cells exhibit characteristics of early chamber myocardium.. J Mol Cell Cardiol.

[33] Boheler KR, Czyz J, Tweedie D, Yang HT, Anisimov SV (2002). Differentiation of pluripotent embryonic stem cells into cardiomyocytes.. Circ Res.

[34] Babai F, Musevi-Aghdam J, Schurch W, Royal A, Gabbiani G (1990). Coexpression of α-sarcomeric actin, α-smooth muscle actin and desmin during myogenesis in rat and mouse embryos.. Differentiation.

[35] Eppenberger-Eberhardt M, Flamme I, Kurer V, Eppenberger HM (1990). Reexpression of α-smooth muscle actin isoform in cultured adult rat cardiomyocytes.. Developmental Biology.

[36] Park KW, Goo JH, Chung HS, Kim H, Kim DH (1998). Cloning of the genes encoding mouse cardiac and skeletal calsequestrins: expression pattern during embryogenesis.. Gene.

[37] Houweling AC, van Borren MM, Moorman AF, Christoffels VM (2005). Expression and regulation of the atrial natriuretic factor encoding gene Nppa during development and disease.. Cardiovasc Res.

[38] Sabry MA, Dhoot GK (1989). Identification and pattern of expression of a developmental isoform of troponin I in chicken and rat cardiac muscle.. JMuscle ResCell Motil.

[39] Saggin L, Gorza L, Ausoni S, Schiaffino S (1989). Troponin I switching in the developing heart.. Journal of Biological Chemistry.

[40] Lam EW, Watson RJ (1993). An E2F-binding site mediates cell-cycle regulated repression of mouse B-myb transcription.. Embo J.

[41] Li J, Wei H, Chesley A, Moon C, Krawczyk M (2007). The pro-angiogenic cytokine pleiotrophin potentiates cardiomyocyte apoptosis through inhibition of endogenous AKT/PKB activity.. J Biol Chem.

[42] Wu SM, Fujiwara Y, Cibulsky SM, Clapham DE, Lien CL (2006). Developmental origin of a bipotential myocardial and smooth muscle cell precursor in the mammalian heart.. Cell.

[43] Christoforou N, Miller RA, Hill CM, Jie CC, McCallion AS (2008). Mouse ES cell-derived cardiac precursor cells are multipotent and facilitate identification of novel cardiac genes.. J Clin Invest.

[44] Franco D, Lamers WH, Moorman AF (1998). Patterns of expression in the developing myocardium: towards a morphologically integrated transcriptional model.. Cardiovasc Res.

[45] Kolossov E, Lu Z, Drobinskaya I, Gassanov N, Duan Y (2005). Identification and characterization of embryonic stem cell-derived pacemaker and atrial cardiomyocytes.. Faseb J.

[46] Miller-Hance WC, LaCorbiere M, Fuller SJ, Evans SM, Lyons G (1993). *In vitro* chamber specification during embryonic stem cell cardiogenesis.. J Biol Chem.

[47] Christoffels VM, Habets PE, Franco D, Campione M, de Jong F (2000). Chamber formation and morphogenesis in the developing mammalian heart.. Dev Biol.

[48] Carrier L, Boheler KR, Chassagne C, de la Bastie D, Wisnewsky C (1992). Expression of the sarcomeric actin isogenes in the rat heart with development and senescence.. Circulation Research.

[49] Kolossov E, Fleischmann BK, Liu Q, Bloch W, Viatchenko-Karpinski S (1998). Functional characteristics of ES cell-derived cardiac precursor cells identified by tissue-specific expression of the green fluorescent protein.. J Cell Biol.

[50] Koban MU, Moorman AF, Holtz J, Yacoub MH, Boheler KR (1998). Expressional analysis of the cardiac Na-Ca exchanger in rat development and senescence.. Cardiovasc Res.

[51] Barth AS, Kizana E, Smith RR, Terrovitis J, Dong P (2008). Lentiviral vectors bearing the cardiac promoter of the Na+-Ca2+ exchanger report cardiogenic differentiation in stem cells.. Mol Ther.

[52] Fijnvandraat AC, van Ginneken AC, de Boer PA, Ruijter JM, Christoffels VM (2003). Cardiomyocytes derived from embryonic stem cells resemble cardiomyocytes of the embryonic heart tube.. Cardiovasc Res.

[53] Cloud JE, Rogers C, Reza TL, Ziebold U, Stone JR (2002). Mutant mouse models reveal the relative roles of E2F1 and E2F3 *in vivo*.. Mol Cell Biol.

[54] Soonpaa MH, Kim KK, Pajak L, Franklin M, Field LJ (1996). Cardiomyocyte DNA synthesis and binucleation during murine development.. Am J Physiol.

[55] Zandstra PW, Bauwens C, Yin T, Liu Q, Schiller H (2003). Scalable production of embryonic stem cell-derived cardiomyocytes.. Tissue Eng.

[56] Sage J, Miller AL, Perez-Mancera PA, Wysocki JM, Jacks T (2003). Acute mutation of retinoblastoma gene function is sufficient for cell cycle re-entry.. Nature.

[57] Shackney SE, Shankey TV (1999). Cell cycle models for molecular biology and molecular oncology: exploring new dimensions.. Cytometry.

[58] MacPherson D, Sage J, Crowley D, Trumpp A, Bronson RT (2003). Conditional mutation of Rb causes cell cycle defects without apoptosis in the central nervous system.. Mol Cell Biol.

[59] Murry CE, Field LJ, Menasche P (2005). Cell-based cardiac repair: reflections at the 10-year point.. Circulation.

[60] Takahashi K, Yamanaka S (2006). Induction of pluripotent stem cells from mouse embryonic and adult fibroblast cultures by defined factors.. Cell.

[61] Koh GY, Soonpaa MH, Klug MG, Pride HP, Cooper BJ (1995). Stable fetal cardiomyocyte grafts in the hearts of dystrophic mice and dogs.. J Clin Invest.

[62] Baker JE, Holman P, Gross GJ (1999). Preconditioning in immature rabbit hearts: role of KATP channels.. Circulation.

[63] Muhlfeld C, Singer D, Engelhardt N, Richter J, Schmiedl A (2005). Electron microscopy and microcalorimetry of the postnatal rat heart (Rattus norvegicus).. Comp Biochem Physiol A Mol Integr Physiol.

[64] Muhlfeld C, Urru M, Rumelin R, Mirzaie M, Schondube F (2006). Myocardial ischemia tolerance in the newborn rat involving opioid receptors and mitochondrial K+ channels.. Anat Rec A Discov Mol Cell Evol Biol.

[65] Wobus AM, Guan K, Yang H-T, Boheler KR, Turksen K (2002). Embryonic stem cells as a model to study cardiac, skeletal muscle and vascular smooth muscle cell differentiation.. Methods Mol Biol.

[66] Boheler KR (2003). ES cell differentiation to the cardiac lineage.. Methods Enzymol.

[67] Takahashi T, Lord B, Schulze PC, Fryer RM, Sarang SS (2003). Ascorbic acid enhances differentiation of embryonic stem cells into cardiac myocytes.. Circulation.

[68] Tarasov KV, Tarasova YS, Tam WL, Riordon DR, Elliott ST (2008). B-MYB is Essential for Normal Cell Cycle Progression and Chromosomal Stability of Embryonic Stem Cells.. PLoS One.

[69] Tarasova YS, Riordon DR, Tarasov KV, Boheler KR, Notarianni E, Evans MJ (2006). *In vitro* differentiation of mouse ES cells to muscle cells.. Embryonic Stem Cells.

[70] Fan JS, Palade P (1998). Perforated patch recording with beta-escin.. Pflugers Arch.

